# Incomplete Sterility of Chromosomal Hybrids: Implications for Karyotype Evolution and Homoploid Hybrid Speciation

**DOI:** 10.3389/fgene.2020.583827

**Published:** 2020-10-15

**Authors:** Vladimir A. Lukhtanov, Vlad Dincă, Magne Friberg, Roger Vila, Christer Wiklund

**Affiliations:** ^1^Department of Karyosystematics, Zoological Institute of Russian Academy of Sciences, Saint Petersburg, Russia; ^2^Ecology and Genetics Research Unit, University of Oulu, Oulu, Finland; ^3^Institut de Biologia Evolutiva (CSIC-Universitat Pompeu Fabra), Barcelona, Spain; ^4^Biodiversity Unit, Department of Biology, Lund University, Lund, Sweden; ^5^Department of Zoology, Stockholm University, Stockholm, Sweden

**Keywords:** chromosome, inverted meiosis, fertility, inviability, hybridization, segregation, Pieridae, Lepidoptera

## Abstract

Heterozygotes for major chromosomal rearrangements such as fusions and fissions are expected to display a high level of sterility due to problems during meiosis. However, some species, especially plants and animals with holocentric chromosomes, are known to tolerate chromosomal heterozygosity even for multiple rearrangements. Here, we studied male meiotic chromosome behavior in four hybrid generations (F1–F4) between two chromosomal races of the Wood White butterfly *Leptidea sinapis* differentiated by at least 24 chromosomal fusions/fissions. Previous work showed that these hybrids were fertile, although their fertility was reduced as compared to crosses within chromosomal races. We demonstrate that (i) F1 hybrids are highly heterozygous with nearly all chromosomes participating in the formation of trivalents at the first meiotic division, and (ii) that from F1 to F4 the number of trivalents decreases and the number of bivalents increases. We argue that the observed process of chromosome sorting would, if continued, result in a new homozygous chromosomal race, i.e., in a new karyotype with intermediate chromosome number and, possibly, in a new incipient homoploid hybrid species. We also discuss the segregational model of karyotype evolution and the chromosomal model of homoploid hybrid speciation.

## Introduction

Chromosomal heterozygosity leads to the formation of multivalents (instead of normal bivalents) during meiosis. Usually, this results in segregation problems at the first meiotic division, production of unbalanced gametes and, as a consequence, complete or partial sterility (Grant, 1981; King, 1993; Borodin et al., 2019). Even a single heterozygous chromosomal rearrangement, such as a reciprocal translocation or chromosomal fusion, is expected to result in 50% reduction of fertility (King, 1993). In the case of heterozygosity for multiple rearrangements, the rate of balanced gametes should decrease strongly and could be as low as 1/2n, where n is the number of heterozygous rearrangements (Grant, 1981). Sometimes, the observed number of sterile and/or inviable gametes can be lower than this expectation due to the orientation of multivalents during meiosis (King, 1993), preferential inclusion of inviable nuclei in polar bodies in females (Cortes et al., 2015; Borodin et al., 2019), lower recombination rates in the heterogametic sex (Lenormand and Dutheil, 2005), or distorting transmission ratios during meiosis caused by selfish genetic elements (Akera et al., 2019). Nevertheless, in general, fertility decreases with increased chromosomal heterozygosity (King, 1993; Castiglia and Capanna, 2000). However, organisms can sometimes tolerate heterozygosity for multiple rearrangements (Lukhtanov et al., 2011; Dobigny et al., 2017), raising questions about additional mechanisms that may rescue fertility in chromosomal hybrids.

One of these additional mechanisms is inverted meiosis (Lukhtanov et al., 2018, 2020). In normal conventional meiosis, the first meiotic division is reductional, resulting in segregation of chromosomal homologs, whereas the second meiotic division is equational, resulting in separation of sister chromatids. Inverted meiosis has an opposite order of these main meiotic events (Lenormand et al., 2016; Loidl, 2016; Archetti, 2020). Usually, inverted meiosis can be found in organisms with holocentric chromosomes (Murakami and Imai, 1974; Melters et al., 2012), which are characterized by kinetic activity distributed along almost the entire chromosome length (Melters et al., 2012; Bures et al., 2013; Heckmann et al., 2014; Manicardi et al., 2015; Houben et al., 2018). Species with holocentric chromosomes occur in multiple phyla of animals and plants (Kuznetsova, 1979; Melters et al., 2012; Bures et al., 2013) and may represent as much as 30% of eukaryotic diversity (Lukhtanov et al., 2018). In recent years, inverted meiosis has been demonstrated for some monocentric chromosomes of humans (Ottolini et al., 2015) and yeast (Lu and He, 2019) indicating that this type of meiosis is more widespread in nature than previously thought.

There is a fundamental difference between canonical and inverted meiosis in the behavior and fate of the chromosomal multivalents. In canonical meiosis, chromosomal heterozygotes are expected to have segregation problems in the first meiotic anaphase, since homologous chromosome pairing is complicated by crossing over. This produces a very intricate multivalent structure, which has a high probability of resulting in unbalanced segregation of genetic material. In inverted meiosis, these problems are avoided because sister chromatids (but not homologs) segregate in the first anaphase, resulting in a balanced transmission of genetic material to metaphase II cells. Thus, the metaphase II multivalents have a simpler structure compared to metaphase I multivalents, and their balanced segregation at anaphase II is more probable (Lukhtanov et al., 2018).

Insects of the order Lepidoptera (i.e., butterflies and moths) have holocentric chromosomes (Murakami and Imai, 1974), and some species demonstrate inverted meiosis (Lukhtanov et al., 2018, 2020), as well as a very high level of inter- and intrapopulation variation in karyotypes (Lukhtanov et al., 2011). Therefore, it is not surprising that the most extreme examples of viable chromosomal hybrids are found in this order (Nagaraju and Jolly, 1986; Traut and Clarke, 1997; Hora et al., 2019).

Laboratory hybrids between two chromosomal races of the Wood White butterfly *Leptidea sinapis* represent a remarkable case (Lukhtanov et al., 2018). This butterfly displays the widest documented intraspecific variability in chromosome number known in eukaryotes, excluding cases of polyploidy (Lukhtanov et al., 2011). Within this species, the diploid chromosome number gradually decreases from 2n = 106, 108 in north-eastern Spain to 2n = 56 in eastern Kazakhstan, and to 2n = 57, 58 in south-eastern Sweden (Lukhtanov et al., 2018). This cline was likely generated due to the secondary contact of two chromosomally diverged populations (Talla et al., 2019). The intraspecific nature of the extreme level of variability in chromosome number within this clade is supported by genetic and morphological data, as well as by mating experiments (Lukhtanov et al., 2011; Dincă et al., 2013). The mating experiments also showed that the hybrids between the Spanish and Swedish races, separated by at least 24 chromosomal fusions/fissions, displayed, contrary to the theoretical prediction, relatively high reproductive fitness (42% of that of the control lines) and regular behavior of meiotic chromosomes (Lukhtanov et al., 2018).

In this work, we demonstrate that (i) F1 hybrids between the Spanish and Swedish races are highly heterozygous with nearly all chromosomes participating in formation of trivalents at the first meiotic division, and (ii) that from F1 to F4 the number of trivalents decreases and the number of bivalents increases. Then, we analyze the evolutionary significance of incomplete, but still relatively high, fertility of chromosomal hybrids in processes such as the generation of new karyotypes and homoploid hybrid speciation (HHS).

## Materials and Methods

### Samples

We analyzed the squashed chromosome preparations of the *L. sinapis* F1–F4 hybrid individuals that were previously used to study inverted meiosis, and hybrid viability and fertility (Lukhtanov et al., 2018). These previous data showed a rather high, albeit reduced, level of fitness as compared to control, chromosomally homozygous Swedish, and Spanish lines (Lukhtanov et al., 2018). Two main laboratory lines were established based on wild-caught individuals, which were identified based on genitalia examination and/or DNA barcoding following standard protocols (Dincă et al., 2011, 2013). One laboratory line was representative for *L. sinapis* populations with high chromosome number (2n = 106, 108) and included specimens originating from north-eastern Spain (Montseny area, Barcelona Province, Catalonia). The other line was representative for *L. sinapis* populations with low chromosome number (2n = 57, 58) and included specimens originating from south central Sweden (two field sites in the vicinity of Stockholm). Mating experiments were performed under laboratory conditions at the Department of Zoology, Stockholm University, Sweden. Pure Spanish and Swedish laboratory lines were maintained and used as controls with respect to crosses between male Spanish and female Swedish *L. sinapis*, and *vice versa*. All possible mating combinations between Spanish and Swedish *L. sinapis* were performed until F2, and each mating combination was represented by at least five different pairs of specimens. The offspring of these pairs were bred to adults on the host plant *Lotus corniculatus* and used for further experiments. For generations F3 and F4, a subset of the potential hybrid mating combinations was performed. The laboratory mating protocol and rearing protocol are described in detail in Lukhtanov et al. (2018). In each generation, a number of larval and adult offspring from each mating combination was sacrificed for karyological studies (Lukhtanov et al., 2018). Apart from the sacrificed larvae, the vast majority of last-instar larvae successfully reached adulthood. Eclosing adults were used in subsequent mating experiments and females were allowed to lay eggs to maintain the laboratory lines. The above-mentioned process was reiterated across forthcoming generations (until F2 for within-population crosses and until larval generation F4 for a subset of hybrid crosses).

### Chromosomal Analysis

Only recently eclosed adult males were used to analyze meiosis and to study meiotic karyotypes. Adults were euthanized by a sharp pinch to the thorax and testes were immediately excised and placed into 0.5-mL vials with freshly prepared Carnoy fixative (ethanol and glacial acetic acid, 3:1). Gonads were stored in fixative for 2–6 months at 4°C and then stained with 2% acetic orcein for 30 days at 20°C. Squashed chromosome preparations were done and cytogenetic analysis was conducted as previously described (Lukhtanov et al., 2019). The diploid chromosome number (2n) was counted in atypical meiosis, which represents a type of asynaptic meiosis and displays a diploid number of chromosomes (see Lorkoviæ, 1990 for a review of atypical meiosis in Lepidoptera).

#### Abbreviations

ca (circa) means that the count was made with an approximation due to insufficient quality of the preparation or overlapping of some chromosomes or bivalents;

HHS is homoploid hybrid speciation;

m is the diploid chromosome number counted in male asynaptic meiosis;

MI is the first meiotic division;

MII is the second meiotic division;

N is effective population size;

n is the total number of observed chromosomal elements at MI or/and MII (except for small dot-like univalents); in fact, it represents the sum of bivalents and trivalents;

s is a small dot-like chromosomal element (most likely univalent).

### Estimation of the Number of Bivalents and Trivalents at the MI Stage

Based on the karyotypes in parental Swedish and Spanish races, we know that in F1 hybrids, MI karyotypes include at least 24 trivalents and several bivalents. Univalents were also sporadically observed in hybrids (especially in F1 hybrids). These different elements can be visually identified: bivalents have a dumbbell shape, trivalents present an irregular configuration and univalents are small dot-like elements. However, the visual distinction between bi- and trivalents on the preparation may be difficult because the shape of the elements strongly depends on their orientation within the cell. Therefore, we used the following approach to calculate the number of bi- and trivalents: First, we counted the number of all visible elements (i.e., bivalents + trivalents, in some cases also univalents) at MI stage. Then, we counted the diploid chromosome number at male asynaptic meiosis.

We applied the formula

2x + 3y = m,

where x is the number of bivalents in meiosis, y is the number of trivalents in meiosis, and m is the diploid number.

We also calculated

x + y = n,

where x is the number of bivalents in meiosis, y is the number of trivalents in meiosis, and n is the total number of elements (except for small dot-like univalents) in meiosis.

Thus, in order to determine the number of bivalents and trivalents in the karyotype, it is necessary to solve this system of two equations with two unknowns.

The solution to this system of equations provides the answer:

*y* = m−2n,

*x* = n−y

where x is the number of bivalents in meiosis, y is the number of trivalents in meiosis, m is the diploid number, and n is the number of elements at MI.

To calculate the proportion of meiotic chromosomes in homozygous state (i.e., part of bivalents, not part of trivalents), we used the formula 2x/m, where x is the number of bivalents in meiosis, and m is the diploid number determined in asynaptic meiosis. We used One-Way ANOVA to test for the effect of generation (1–4) on this parameter. The significance of the pairwise differences was then tested with an Unequal N HSD test.

## Results

### Karyotype of the Parental Lines

**FIGURE 1:**
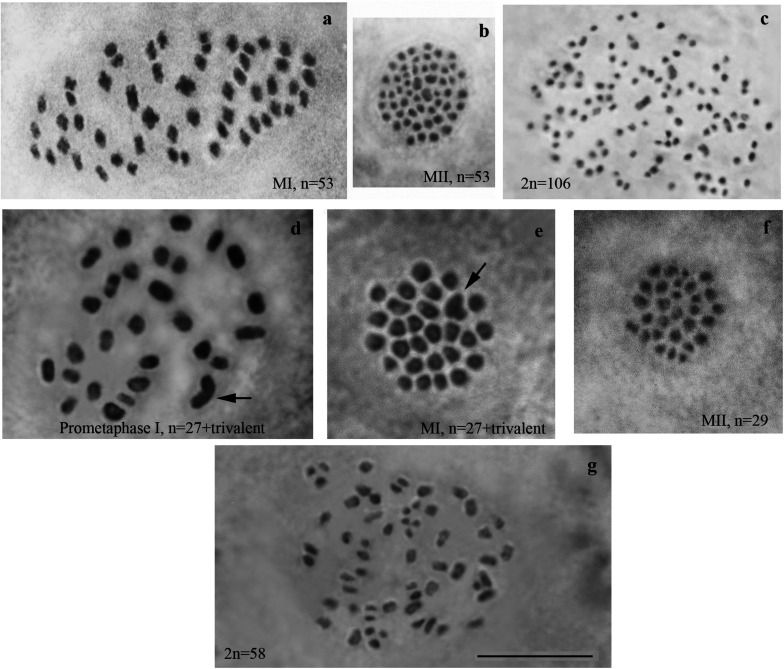
Karyotypes of Spanish and Swedish parental lines of *Leptidea sinapis*. Putative trivalents are indicated by arrows. Scale bar = 10 μm. **(a)** Spain, sample 07F568, MI, *n* = 53. **(b)** Spain, sample 07C470, MII, *n* = 53. **(c)** Spain, sample 08H275, 2n = 106. **(d)** Sweden, sample 12M060, prometaphase I, *n* = 27 + trivalent. **(e)** Sweden, sample 12M058, MI, *n* = 27 + trivalent. **(f)** Sweden, sample 12Z085, MII, *n* = 29. **(g)** Sweden, sample 10B467, asynaptic meiosis, 2n = 58.

We studied karyotype preparations of six samples of *L. sinapis* from north-eastern Spain and seven samples of *L. sinapis* from Sweden (Figure 1). Four Spanish samples were used in a previous study (Lukhtanov et al., 2011) and were reanalyzed. Two samples, representing the Spanish laboratory line, were analyzed for the first time. In five of the Spanish samples, at the MI stage, 53 chromosomal elements were observed and all of them were most likely bivalents. At the MII stage, 53 chromosomes were observed. The diploid chromosome number was counted to be 2n = 106. In one sample the chromosomal elements were counted with approximation due to overlapping of some elements (*n* = ca53–54, 2n = ca106–108). In the Swedish samples, either 28 or 29 elements were observed at MI and MII, and 57 or 58 elements were observed in male asynaptic meiosis. In specimens with *n* = 28, 27 elements were bivalents, and one element was interpreted as trivalent due to its irregular shape. In specimens with *n* = 28, 57 chromosomes were counted in male asynaptic meiosis. According to our formulas (*y* = m−2n, *x* = n−y) these samples have 27 bivalents and one trivalent at MI. Thus, this calculation confirms the interpretation of chromosomal elements based on their morphology. In specimens with *n* = 29, 58 chromosomes were counted in male asynaptic meiosis. All MI elements were interpreted as bivalents in these samples. Based on counts and morphology of chromosomal elements, we conclude that the Swedish population is heterozygous for a single chromosome fusion/fission resulting in variation in chromosome number.

### F1 Karyotype of Chromosomal Race Hybrids

**FIGURE 2:**
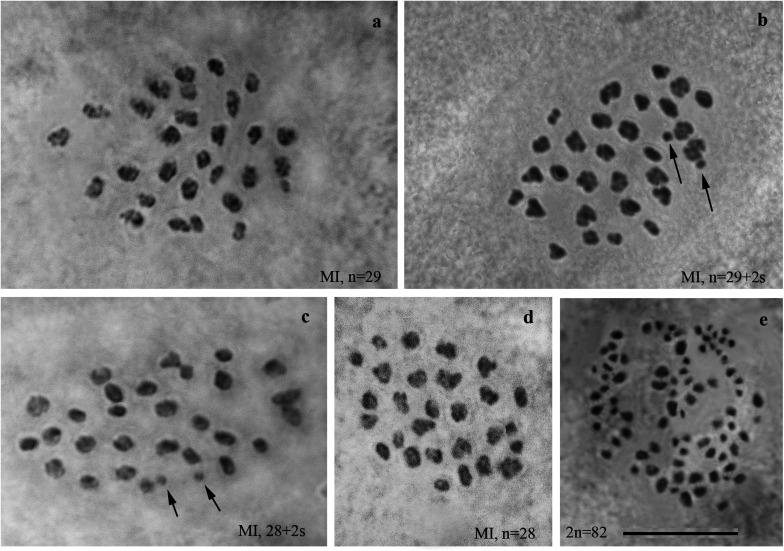
Karyotypes of F1 hybrids between Spanish and Swedish chromosomal races of *Leptidea sinapis*. Univalents are indicated by arrows. Scale bar = 10 μm. **(a)** sample 12Z052, MI, *n* = 29. **(b)** sample12Z065, MI, *n* = 29 + 2s. **(c)** sample 12Z052, MI, *n* = 28 + 2s. **(d)** sample 13Y058, *n* = 28. **(e)** sample 13Y039, asynaptic meiosis, 2n = 82.

We studied the karyotype preparations of twelve F1 samples (Figure 2). In eight samples, between 28 and 32 elements were observed at MI and MII, and in ten samples, 81 or 82 chromosomes were counted in male asynaptic meiosis (Table 1). In seven of the eight samples, 29 elements were observed at MI as a single or a modal chromosomal count, and in one sample *n* = 28 was counted. Most of these elements had irregular shape and could be interpreted as trivalents. In four of the eight samples, the number of chromosomal entities at MI was the same (28 or 29) as in the parental Swedish population indicating perfect or nearly perfect conjugation of the 53 smaller chromosomes of Spanish origin with the 28 or 29 bigger chromosomes of Swedish origin. In the four other samples, 1–3 additional tiny dot-like chromosomal univalents were observed, most likely indicating an imperfect meiotic pairing. According to our formulas (see “Materials and Methods”), the F1 samples had between 3 and 5 bivalents and 24–25 trivalents.

**TABLE 1:** Chromosome numbers of the studied specimens of *Leptidea sinapis*.

Sample ID	Line	Number of elements observed at MI (n)	Number of chromosomes in male asynaptic meiosis (m)	Estimated number of bivalents *x* = n−y	Estimated number of trivalents *y* = m−2n
08H275	Spain	–	106	53	0
08H281	Spain	–	106	53	0
07C470	Spain	53 (at MII)	–	53	0
07F568	Spain	53	–	53	0
L1	Spain	53	ca106	53	0
L2	Spain	–	ca106–108	ca53–54	0
L3	Sweden	–	57	unknown	unknown
L4	Sweden	–	58	unknown	unknown
12M060	Sweden	28	57	27	1
12M058	Sweden	28	57	27	1
12M049	Sweden	29	–	29	0
12Z085	Sweden	29	58	29	0
10-B467	Sweden	29	ca58	29	0
13Y025	F_1_ hybrid	–	82	unknown	unknown
13Y037	F_1_ hybrid	–	82	unknown	unknown
13Y050	F_1_ hybrid	–	82	unknown	unknown
13Y039	F_1_ hybrid		82		
13Y058	F_1_ hybrid	28	ca81	3	25
12Z065	F_1_ hybrid	29 + 2s	ca82	5	24
13Y045	F_1_ hybrid	29	ca82	5	24
13Y057	F_1_ hybrid	29	–	unknown	unknown
13Y063	F_1_ hybrid	29	ca82	5	24
12Z066	F_1_ hybrid	29, 29 + s, 29 + 2s, 29 + 3s	ca82	5	24
12Z051	F_1_ hybrid	29 + s, 29 + 2s	ca82	5	25
12Z054	F_1_ hybrid	ca29 + s, ca29 + 2s, ca29 + 3s	–	unknown	unknown
L7	F_2_ hybrid	–	ca73	unknown	unknown
L8	F_2_ hybrid	–	ca77	unknown	unknown
L9	F_2_ hybrid	–	ca77	unknown	unknown
L10	F_2_ hybrid	–	ca82	unknown	unknown
L11	F_2_ hybrid	–	ca82	unknown	unknown
L12	F_2_ hybrid	–	83	unknown	unknown
L13	F_2_ hybrid	–	83	unknown	unknown
L14	F_2_ hybrid	–	83	unknown	unknown
L15	F_2_ hybrid	–	85	unknown	unknown
L16	F_2_ hybrid	–	ca90	unknown	unknown
13Y080	F_2_ hybrid	31	ca82	11	20
11H440	F_2_ hybrid	32	82	14	18
13Y079	F_2_ hybrid	32	ca77	19	13
13Y082	F_2_ hybrid	32	ca81	15	17
11H437	F_2_ hybrid	32	–	unknown	unknown
13Y084	F_2_ hybrid	32, 32 + s	82	14	18
13Y083	F_2_ hybrid	34	ca83	19	15
11H479	F_2_ hybrid	35	ca87	18	17
13Y077	F_2_ hybrid	35, 35 + s	ca82	23	12
11H467	F_2_ hybrid	35	ca83	22	13
13Y081	F_2_ hybrid	36	ca85	23	13
11H439	F_2_ hybrid	36	–	unknown	unknown
L20	F_3_ hybrid	–	76	unknown	unknown
L21	F_3_ hybrid	–	76	unknown	unknown
L22	F_3_ hybrid	–	77	unknown	unknown
L23	F_3_ hybrid	–	86	unknown	unknown
14A002	F_3_ hybrid	29	ca75	12	17
14A004	F_3_ hybrid	29	ca74	13	16
14A003	F_3_ hybrid	31	ca77	16	15
14A001	F_3_ hybrid	32	ca76	20	12
14A000	F_3_ hybrid	33	ca82	17	16
14A005	F_3_ hybrid	35	ca84	21	14
14B000	F_4_ hybrid	32, 32 + s	ca79	17	15
14B003	F_4_ hybrid	33	ca78	21	12
14B004	F_4_ hybrid	33	ca82	17	16
14B002	F_4_ hybrid	34	ca83	19	15
14B005	F_4_ hybrid	37	ca86	25	12
14B001	F_4_ hybrid	38	ca89	25	13

### F2 Karyotype of Chromosomal Race Hybrids

**FIGURE 3:**
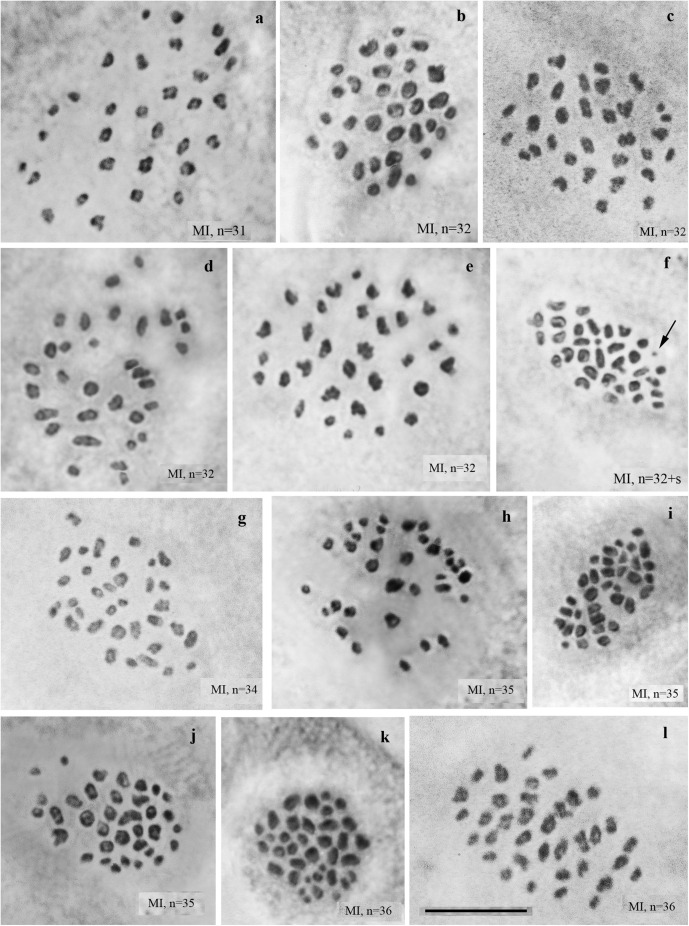
MI karyotypes of F2 hybrids between Spanish and Swedish chromosomal races of *Leptidea sinapis*. The univalent is indicated by an arrow. Scale bar = 10 μm. **(a)** sample 13Y080, *n* = 31. **(b)** sample 11H440, *n* = 32. **(c)** sample 13Y079, *n* = 32. **(d)** sample 13Y082, *n* = 32. **(e)** sample 13Y084, *n* = 32. **(f)** sample 13Y084, *n* = 32 + s. **(g)** sample 13Y083, *n* = 34. **(h)** sample 11H467, *n* = 35. **(I)** sample 11H479, *n* = 35. **(j)** sample 13Y077, *n* = 35. **(k)** sample 11H439, *n* = 36. **(l)** sample 13Y081, *n* = 36.

We studied the karyotype preparations of 22 F2 samples (Figure 3). In these samples, between 31 and 36 elements were observed at MI and MII, and 73 to ca 90 chromosomes were observed in male asynaptic meiosis (Table 1). Thus, the number of entities was very variable in both haploid and diploid stages of meiosis. Many MI elements had irregular shape and can be interpreted as trivalents. In two samples, an additional tiny dot-like chromosomal univalent was observed indicating an imperfect meiotic pairing, but such unpaired elements were rarer than in F1 hybrids. According to our formulas (see “Materials and Methods”), the F2 samples had between 11 and 23 bivalents and 12–20 trivalents.

### F3 Karyotype of Chromosomal Race Hybrids

**FIGURE 4:**
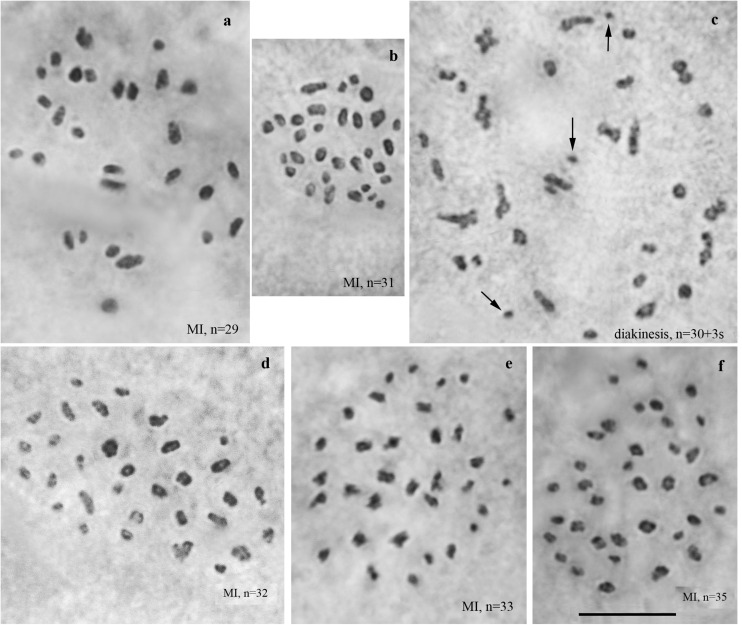
Karyotypes of F3 hybrids between Spanish and Swedish chromosomal races of *Leptidea sinapis*. Univalents are indicated by arrows. Scale bar = 10 μm. **(a)** sample 14A004, MI, *n* = 29. **(b)** sample 14A003, MI, *n* = 31. **(c)** sample 14A003, diakinesis, *n* = 30 + 3s. **(d)** sample 14A001, MI, *n* = 32. **(e)** sample 14A000, MI, *n* = 33. **(f)** sample 14A005, MI, *n* = 35.

We studied the karyotype preparations of ten F3 samples (Figure 4). In these samples, between 29 and 35 elements were observed at MI and MII. Many MI elements (most likely trivalents) had irregular shape. From 74 to 86 chromosomes were observed at male asynaptic meiosis (Table 1). According to our formulas (see “Materials and Methods”), the F3 samples had between 12 and 21 bivalents and 12–17 trivalents.

### F4 Karyotype of Chromosomal Race Hybrids

**FIGURE 5:**
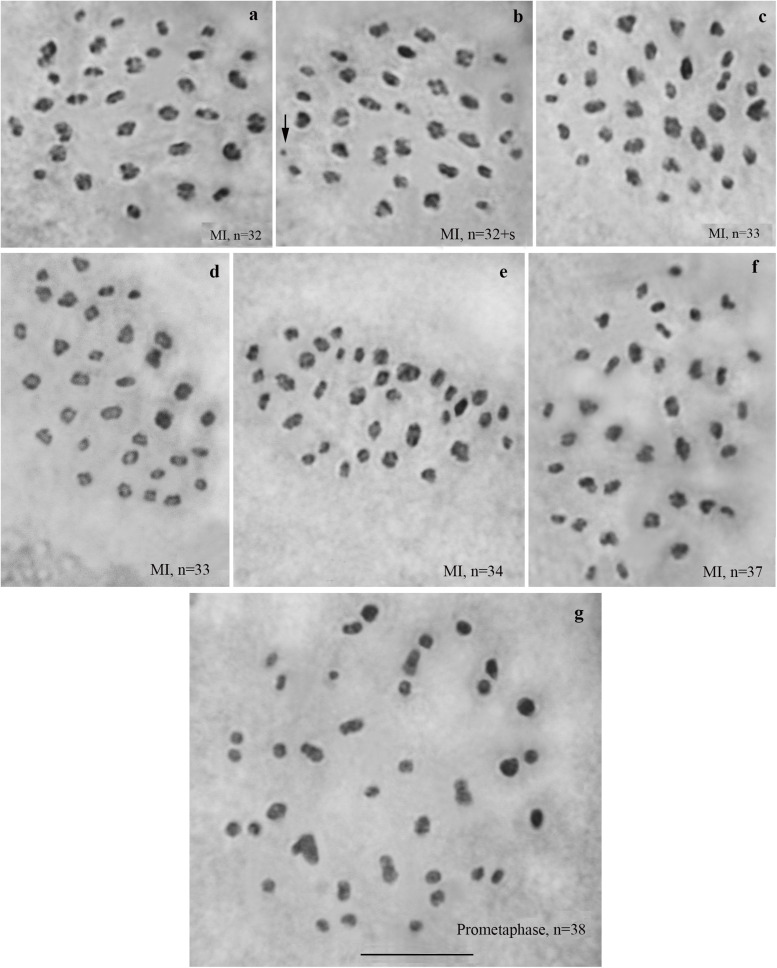
Karyotypes of F4 hybrids between Spanish and Swedish chromosomal races of *Leptidea sinapis*. The univalent is indicated by an arrow. Scale bar = 10 μm. **(a)** sample 14B000, MI, *n* = 32. **(b)** sample 14B000, MI, *n* = 32 + s. **(c)** sample 14B003, MI, *n* = 33. **(d)** sample 14B004, MI, *n* = 33. **(e)** sample 14B002, MI, *n* = 34. **(f)** sample 14B005, MI, *n* = 37. **(g)** sample 14B001, prometaphase I, *n* = 38.

We studied the karyotype preparations of six F4 samples (Figure 5). In these samples, between 32 and 38 elements were observed at MI and MII; some of these elements (most likely trivalents) had irregular shape and configuration. From ca 79 to ca 89 chromosomes were observed at male asynaptic meiosis (Table 1). According to our formulas (see “Materials and Methods”), the F4 samples had between 17 and 25 bivalents and 12–16 trivalents.

### Dynamics in the Level of Chromosomal Homozygosity From F1 to F4

Based on the data in Table 1, we calculated the proportions of chromosomes in homozygous state (i.e., parts of bivalents, not parts of trivalents) in each examined individual and in each generation (Table 2). The effect of generation on this parameter was found to be highly significant (*p* < 0.001). The Unequal N HSD test showed that only the first generation significantly differed from the others in the proportion of chromosomes in homozygous state (*p* < 0.001). All other pairwise differences were non-significant, although the highest mean proportion of chromosomes in homozygous state was found in F4.

**TABLE 2:** Dynamics in the level of chromosomal homozygosity from F1 to F4.

	Number of bivalents (range)	Number of trivalents (range)	Proportion of chromosomes in homozygous state (range),%	Proportion of chromosomes in homozygous state (mean ± SD),%
F1	3–5	24–25	7.4–12.2	11.2 ± 2.14
F2	11–23	12–20	26.8–56.1	43.2 ± 10.00
F3	12–21	12–17	32.0–52.6	42.1 ± 8.06
F4	17–25	12–16	43.0–58.1	49.7 ± 7.18

## Discussion

### Hybrid Karyotype and Tendency to Homozygosity Across Generations

Since the karyotypes of the parental Spanish and Swedish races of the Wood White butterfly *L. sinapis* are known (Lukhtanov et al., 2011, 2018; this study) and differ by at least 24 chromosomal fusions/fissions, we expected at least 24 trivalents and several (3–5) bivalents at MI, in the first generation (F1) of hybrids. Such a karyotype was found in most F1 individuals and cells, although a higher number of chromosomal entities was sometimes observed among meiotic metaphase I cells within a single specimen due to the presence of univalents, possibly indicating an imperfect meiotic pairing. Due to the almost complete pairing of the smaller chromosomes of the Spanish race (*n* = 53), with the larger chromosomes of the Swedish race (*n* = 28, 29), the number of distinct entities (trivalents + bivalents) observed at MI in the hybrid F1 karyotype was equivalent to that of the Swedish parental karyotype.

The surviving F2 hybrids showed a strong significant increase in the proportion of bivalents and a decrease in the proportion of trivalents, and the surviving hybrids F3 and F4 showed a tendency to a slight gradual increase in the proportion of chromosomes in homozygous state (Table 2). Thus, our data do not provide any evidence suggesting a reversal toward one of the parental karyotypes in the hybrids, as reported for the *Antheraea* silkworm and *Pelophylax* frog interspecific hybrids (Nagaraju and Jolly, 1986; Dedukh et al., 2020). Rather, hybrid karyotypes were intermediate between the parental forms, both in the diploid number of chromosomes in the set and in the number of elements observed at MI across generations.

In our study, we continued the crossbreeding experiments up to the fourth hybrid generation. Considering that the studied F1 to F4 hybrids showed stable fertility at almost half the level of the within-karyotype race crosses (Lukhtanov et al., 2018), we expect that the following generations would have been viable and substantially fertile as well. Under the neutral model (i.e., if chromosomal heterozygosity does not affect fitness), the proportion of heterozygotes in the hybrid population will gradually decrease due to genetic drift, similarly to neutral gene alleles (Kimura, 1968). Since the average fixation time of a neutral allele is 4N (where N is the effective population size) (Kimura and Ohta, 1969), after 4N generations we expect that most of the chromosomal rearrangements would be fixed in homozygous state. Thus, it can be expected that, if the process of breeding the hybrid race was continued, a new homozygous karyotype would be obtained, probably with an intermediate number of chromosomes.

In our experiment, the effective population size (i.e., the number of males and females participating in breeding, N) varied from 8 to 40 individuals in different generations (8, 12, 24, 40; *N* = harmonic mean = 15; Lukhtanov et al., 2018, Supporting Information). Thus, it can be expected that in the absence of selection, approximately 4N = 60 generations would be required for the majority of chromosomal alleles to be fixed in homozygous condition under genetic drift. The signs of this transition can already be observed in F2–F4, which show a greater number of bivalents than in F1. This process would be accelerated by a strong inbreeding, when only one pair of individuals is left in each of the generations for reproduction (*N* = 2). Under such conditions, it would take only eight generations before a homozygous or almost homozygous karyotype would be expected to appear.

It should be stressed that *Leptidea* hybrids do not apparently follow a neutral model of evolution, thus our estimates should be interpreted as maximum time to fixation. In fact, given that fitness of the hybrid specimens was 42% as compared to the control pure lines, most likely due to selection against chromosomal heterozygotes, we can expect a higher rate of transition from complete heterozygosity for chromosomal fusions/fissions to a nearly complete homozygosity.

We should also note that Nagaraju and Jolly (1986) observed the reversal to one of the parental karyotypes after thirty generations breeding of *Antheraea* interspecific hybrids, most likely due to selection against the other parental karyotype. In the case of *Leptidea*, such a reversal cannot be entirely ruled out, although its probability seems to be extremely low, based on the data and observations that we have.

### Formation of a Novel Karyotype With a New Diploid Number Through Interspecific Hybridization: The Segregational Model

New karyotypes most often originate because of chromosomal changes and subsequent fixation of novel chromosomal rearrangements in a homozygous state, or through duplication, either of individual chromosomes or of the entire chromosomal set (King, 1993; Schubert and Lysak, 2011). However, there is a third model, which is based on meiotic segregation of chromosomes in interspecific hybrids obtained from cytogenetically differentiated parents.

This model is grounded on works of several influential early geneticists (Müntzing, 1930; Stebbins, 1957; Grant, 1958) who postulated that the sorting of chromosomal rearrangements in hybrid offspring could, by chance, lead to the formation of new population systems that were homozygous for a unique combination of chromosomal sterility factors. By analogy with this scheme, it can be assumed that if two species differentiated by several fixed chromosomal rearrangements hybridize, then, following an initial highly heterozygous stage, a novel homozygous chromosomal complement will be sorted out in the descendants as a result of chromosome segregation. Each of the chromosomal rearrangements fixed in the hybrid lineage is inherited from one of the progenitors, but their combination differs from those in the parental forms. In fact, this scenario of karyotype evolution was explicitly described by Rieseberg, 1997 (and repeated by Coyne and Orr, 2004) in relation to reciprocal translocations.

**FIGURE 6:**
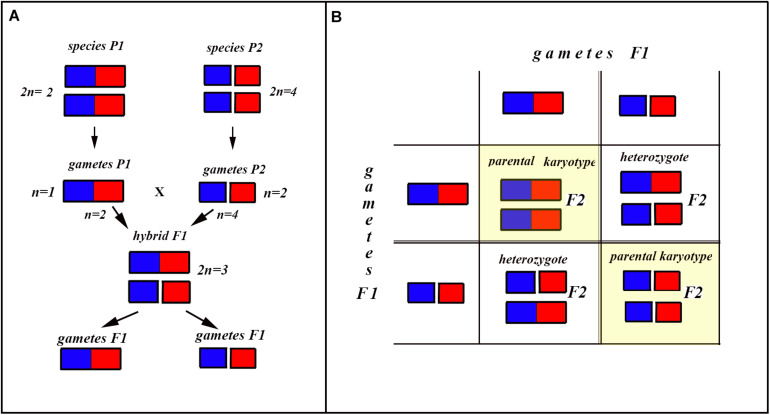
Process and consequence of hybridization between two species differentiated by a single chromosomal fusion/fission. **(A)** Schematic representation of hybridization and formation of gametes. **(B)** The Punnett square predicting the karyotypes of the F2 hybrids. The yellow diagonal indicates the chromosomally homozygous karyotypes. See further explanation in the text.

**FIGURE 7:**
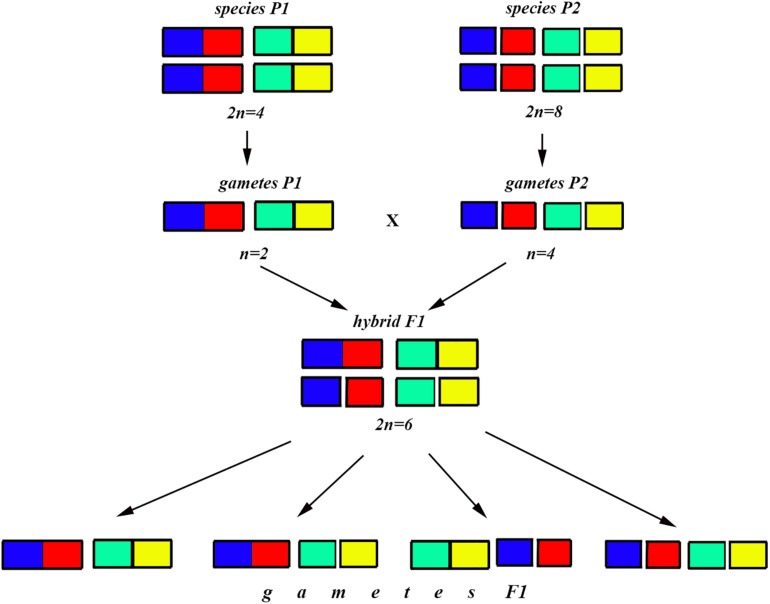
Process and consequence of hybridization between two species differentiated by two chromosomal fusions/fissions: schematic representation of hybridization and formation of gametes.

**FIGURE 8:**
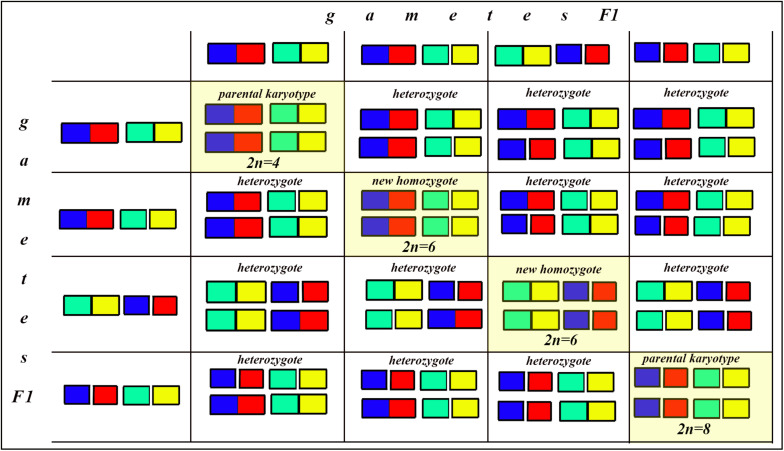
Process and consequence of hybridization between two species differentiated by two chromosomal fusions/fissions: the Punnett square predicting the karyotypes of the F2 hybrids. The yellow diagonal indicates the chromosomally homozygous karyotypes. See further explanation in the text.

In the classical scheme (Rieseberg, 1997; Coyne and Orr, 2004), the number of chromosomes does not change during the formation of a new hybrid chromosomal homozygote. However, if the scheme is applied to fusions or fissions, the number of chromosomes can change (Figures 6–8). Let us first consider an example of hybridization of chromosomal forms, which does not lead to the formation of a new karyotype. If two diploid species P1 and P2 differ by a single chromosomal fusion/fission and hybridize, this will result in a hybrid heterozygous for this single chromosomal fusion/fission (i.e., possessing a trivalent). The F1 hybrid will produce two different types of viable gametes (Figure 6A). These two types of F1 gametes can generate three types of F2 offspring (Figure 6B): two types reconstitute the parental karyotypes P1 and P2, and the third type is the chromosomal heterozygote possessing the same trivalent that was observed in the F1 hybrid. Thus, no new karyotype is created as a consequence of hybridization, and in F2 we observe a reversion to the parental forms.

The situation is entirely different if the parental diploid species differ by two or more chromosomal fusions/fissions. When the F1 hybrid is heterozygous for two chromosomal fusions/fissions (i. e., possesses two trivalents), the F1 hybrid will produce four different types of viable gametes (Figure 7). These four types of F1 gametes will produce 16 different types of F2 offspring (Figure 8). The majority of these types will represent heterozygous karyotypes (all the cells of the Punnett square except for the yellow diagonal). Two types (the first and the last cells of the Punnett square) will reconstitute the parental karyotypes P1 and P2. Interestingly, the two other cells on the diagonal of the Punnett square represent completely new types of chromosomal homozygotes that did not exist in the parental races. These new homozygotes have an intermediate diploid chromosome number 2n = 6, different from both parental 2n = 4 and 2n = 8. Thus, the hybridization led to the formation of homozygous karyotypes with a novel chromosome number. If these F2 offspring are fertile and establish a new breeding population, isolated from the parental races, under a no-selection scenario (i.e., the fusions/fissions are not underdominant) in 4N generations all or nearly all heterozygotes are expected to be eliminated from the population by means of genetic drift. As a result, one of the four homozygous combinations from the “yellow diagonal” will be fixed.

Next, let us consider a more general situation when two parental diploid species differ by *n* chromosomal fusions/fissions. Under this scenario, each trivalent can result in two balanced variants of chromosome segregation. In the case of *n* trivalents and independence of chromosome segregation in each of the trivalents, each balanced gamete variant has a frequency of 1/2^*n*^ (as a probability of a random combination of *n* events, each with a probability of 1/2), and the total number of balanced gametes is 2^*n*^.

After fusing gametes, as it follows from the structure of the Punnett square, the homozygous zygotes form a diagonal (highlighted by yellow in Figure 8 in which the number of cells is equal to the number of gamete variants). In this diagonal, only the first and last cells represent a return to the parental karyotypes. Thus, the proportion of novel possible homozygous combinations (r) can be calculated by the formula: *r* = 1−2/2^*n*^.

That is, if *n* = 1, then *r* = 0

If *n* = 2, then *r* = 0.5

If *n* = 3, then *r* = 0.75

If *n* = 4, then *r* = 0.875

If *n* = 5, then *r* = 0.9375

If *n* = 6, then *r* = 0.96875

…

If *n* = 24 (that is our case), then *r* = 0.99999988.

That is, if two parental species are differentiated by at least three chromosomal fusions/fissions, the probability for the appearance of a new homozygous karyotype in F2 becomes greater than the probability of reversal to one of the parental forms. If two parental species are differentiated by six and more chromosomal fusions/fissions, the reversion becomes an extremely unlikely event compared with the formation of a new homozygous karyotype. This means that, if the chromosomal fusions/fissions are not strongly underdominant (and that is exactly the situation we have in *L. sinapis*) and there is no gene flow from the parental races, the formation of a new homozygous karyotype following hybridization represents the rule rather than the exception.

### Karyotype Evolution and Chromosomal Model of Homoploid Hybrid Speciation

Homoploid hybrid speciation represents a process by which a new reproductively isolated, sexually reproducing species, arises through hybridization and combination of parts of the parental genomes, but without an increase in ploidy (Rieseberg et al., 1995; Rieseberg, 1997; Coyne and Orr, 2004; Mallet, 2007). HHS is frequently defined as “hybrid speciation without change in chromosome number” (Schwarz et al., 2005; Gompert et al., 2006; Mavarez and Linares, 2008; Melo et al., 2009; Salazar et al., 2010; Abbott et al., 2013; Schumer et al., 2014). However, this definition does not reflect the essence of the process and is not accurate because the diploid number of chromosomes may drastically change during HHS (Lukhtanov et al., 2015).

There are two main scenarios of HHS: (i) the scenario based on the formation of pre-zygotic reproductive isolation between the incipient hybrid and the parental species, and (ii) the scenario based on the formation of post-zygotic reproductive isolation between the incipient hybrid and the parental species. The first (“pre-zygotic”) scenario assumes the formation of novel recombinant phenotypes. These phenotypes allow hybrid species to colonize niches unavailable to parental species (Buerkle et al., 2000; Gross and Rieseberg, 2005; Schwarz et al., 2005; Gompert et al., 2006; Kuusela et al., 2007; Hermansen et al., 2010; Stemshorn et al., 2011), and/or to reject parental species as potential mates (Mavarez et al., 2006; Melo et al., 2009; Salazar et al., 2010; Amaral et al., 2014; Hermansen et al., 2014; Barrera-Guzmán et al., 2018; Lamichhaney et al., 2018). The second (“post-zygotic”) scenario assumes the formation of post-zygotic reproductive isolation in the hybrid offspring through sorting of chromosomes (Rieseberg et al., 1995; Lukhtanov et al., 2015; Leducq et al., 2016) and gene alleles (Hermansen et al., 2014; Schumer et al., 2015; Blanckaert and Bank, 2018) resulting in genome or gene incompatibility between the incipient hybrid and both parental species. According to the chromosome-based version of the second scenario (=chromosomal model), two species differentiated by several fixed chromosomal rearrangements hybridize, and then, following an initial heterozygous stage, a new population system is formed that is homozygous for a novel combination of chromosomal rearrangements (Rieseberg, 1997; Coyne and Orr, 2004). Thus, under such a model, speciation is a consequence of the formation of a new recombinant karyotype (Lukhtanov et al., 2015).

The plausibility of the chromosomal model of HHS was shown in laboratory experiments on interspecific hybridization of some plants, where parents were artificially selected (reviewed by Coyne and Orr, 2004: 343-344). This model has also been studied in plants of the genus *Helianthus* (Rieseberg, 1997; Goulet et al., 2017). In animals, it was demonstrated in studies conducted on the butterfly subgenus *Agrodiaetus* (Lukhtanov et al., 2015). The latter study also demonstrated that the diploid chromosome number in hybrid species is drastically different from those in both parental forms if these are differentiated by multiple chromosome fusions/fissions. This model was also supported by findings in hybrid mammals (Giménez et al., 2016) and yeasts (Leducq et al., 2016).

Chromosomal rearrangements can have a dual effect on the generation and maintenance of reproductive barriers. On the one hand, they can suppress recombination in chromosomal hybrids, reducing gene flow between daughter and parental species (a suppressed-recombination mechanism). On the other hand, they can lead to hybrid sterility (a hybrid-sterility mechanism) (Faria and Navarro, 2010). Both of these mechanisms received empirical support (Faria and Navarro, 2010; Luo et al., 2018) and can play a role in HHS. However, much less is known about how a new homozygous karyotype evolves from chromosomal heterozygotes resulting from hybridization. In our study, we experimentally reproduced the first stages of the process potentially leading to chromosome-based HHS. We found that (i) F1 hybrids between chromosomally diverged populations of *L. sinapis* are highly heterozygous with nearly all chromosomes participating in the formation of trivalents at the first meiotic division, and (ii) that there is a tendency of decrease in the number of trivalents and increase in the number of bivalents in subsequent F2, F3, and F4 hybrids. We argue that the observed process of chromosome sorting could, if continued, result in a new homozygous chromosomal race, i.e., in a new karyotype with intermediate chromosome number and, possibly, in a new incipient homoploid hybrid species.

### Chromosomal Hybrids in Laboratory and Chromosomal Cline in Nature: On the Way to HHS

The *L. sinapis* butterfly displays a chromosomal cline with diploid chromosomal numbers ranging from 2n = 106, 108 in Spain to 2n = 56 in Kazakhstan, and to 2n = 57, 58 in Sweden (Lukhtanov et al., 2018). This cline was most likely produced due to the secondary contact of two chromosomally diverged parental populations (Talla et al., 2019). The laboratory experimental data we obtained make it possible to advance hypotheses about how this cline originated and is maintained. Most likely, the original parental races evolved in allopatry – in the Iberian (2n = 106–108) and East European-Asian (2n = 57–58) refugia. Secondary contact in central Europe led to the formation of a hybrid population in which initially the overwhelming majority of individuals were heterozygotes for multiple chromosomal fusions, as this is observed in the F1 hybrids in the laboratory.

Usually, the width of a hybrid zone is determined by the balance between dispersal and selection: low hybrid fertility, resulting in high selection against hybrids, and low dispersal rate lead to a narrow hybrid zone, and *vice versa*, high hybrid fertility and high dispersal rate lead to a wide hybrid zone (Barton and Hewitt, 1985). Since almost half of the *L. sinapis* hybrids are fertile (Lukhtanov et al., 2018), and butterflies, like other winged insects, are mobile, this hybrid zone was likely wide, filling all the space of central Europe during a postglacial expansion phase. Then, for many years after the origin of the hybrid population, two processes acted in parallel – (i) chromosome sorting, leading to the appearance of a homozygous karyotype with an intermediate number of chromosomes and (2) repeated hybridizations with neighboring populations. This led to the formation of a chain of chromosomal forms, between which there are no complete geographic barriers, but which are partially isolated by distance.

Within each semi-isolated chromosomal race, the process of transition to a homozygous karyotype, although close to completion, is still ongoing and chromosomal heterozygotes are common (Lukhtanov et al., 2011). Theoretically, each of these chromosomal races could eventually evolve into a separate species, and the presence of chromosomal differences should contribute to a faster evolution of reproductive isolation. However, this has not yet happened. In fact, we are currently observing an intermediate state in this process, where there is still an influence of the parental forms in the form of gene flow, albeit affected by isolation-by-distance.

In both pre- and post-zygotic models of HHS, the incipient hybrid species, in order to complete speciation, requires a stage of spatial (allopatry) (Gompert et al., 2006) or temporal (allochrony) (Masello et al., 2019) isolation from the parental forms. In the case of *L. sinapis*, complete spatial isolation of the intermediate forms at present may be relatively difficult, given that this butterfly is a common and widespread generalist species (Friberg et al., 2013), and allochrony would be unlikely because it is a multivoltine species in the wide contact zone of central Europe (Friberg et al., 2008). However, it is not impossible to imagine that the species distribution becomes fragmented in the long run, because of human-mediated habitat alteration or climate change, potentially resulting in future speciation.

### Applicability of Segregational Model of Karyotype Evolution and Chromosomal Model of HHS Outside Holocentric Taxa and for Other Types of Chromosomal Rearrangements

The segregational model of karyotype evolution and the chromosomal model of HHS are based on incomplete sterility of chromosomal hybrids. Partial fertility allows a novel combination of chromosomal fusions and fissions to be fixed in a population, and partial sterility acts as a reproductive barrier between the novel hybrid and the parental species. The model organism of our study (*Leptidea sinapis*) is a species with holocentric chromosomes. Such organisms, due to the special organization of their chromosomes, can tolerate heterozygosity even for multiple chromosomal rearrangements (Lukhtanov et al., 2018). Therefore, it can be hypothesized that organisms with holocentric chromosomes will be especially prone to chromosome-based HHS, as has been shown, for example, for the blue butterfly *Polyommatus peilei* (Lukhtanov et al., 2015). In this context, the question arises whether this model is applicable to monocentric organisms. The data available in literature show that, although to a lesser extent, organisms with monocentric chromosomes can also be tolerant to heterozygosity for multiple chromosomal fusions and fissions (e.g., Nachman and Myers, 1989; Nunes et al., 2011; Matveevsky et al., 2020). This indicates the potential of the discussed mechanism for karyotype evolution and speciation in monocentric organisms. Moreover, there is direct evidence that this mechanism produced a new chromosomal race of the monocentric species *Mus musculus* (Giménez et al., 2016). Therefore, it is likely that the proposed model can be applied to both holocentric and monocentric organisms, as long as sterility is incomplete.

The segregational model of karyotype evolution and the chromosomal model of HHS work through the standard mechanism of chromosome segregation, which is found in both canonical and inverted meiosis. These models are not based on the mechanism of inverted meiosis; however, the latter phenomenon can contribute to the formation of new recombinant karyotypes. By increasing the fertility of chromosomal heterozygotes (Lukhtanov et al., 2018), inverted meiosis increases the probability that the chromosome sorting process will be completed, that is, a new homozygous recombinant karyotype is formed.

These models are universal with regard to chromosomal rearrangements and may include not only translocations (Rieseberg, 1997; Coyne and Orr, 2004) and chromosomal fusions and fissions (Lukhtanov et al., 2015; this study), but also other rearrangements (e.g., inversions). As long as the parental species are differentiated by two or more chromosomal rearrangements of any type, then it is theoretically possible that sorting of chromosomes in hybrids can lead to a novel homozygous combination.

## Data Availability Statement

All datasets presented in this study are included in the article.

## Author Contributions

VL: conceptualization, chromosomal analyses, and writing – original draft preparation. VD, MF, RV, and CW: experimental crosses and writing – review and editing. All authors contributed to the article and approved the submitted version.

## Conflict of Interest

The authors declare that the research was conducted in the absence of any commercial or financial relationships that could be construed as a potential conflict of interest.
